# Feel the Fear and Do It Anyway—Beliefs About Compassion Predict Care and Motivation to Help Among Healthcare Professionals

**DOI:** 10.1111/jocn.17477

**Published:** 2024-10-24

**Authors:** Alina Pavlova, Claire O'Donovan‐Lee, Sarah‐Jane Paine, Nathan S. Consedine

**Affiliations:** ^1^ Department of Psychological Medicine University of Auckland Auckland New Zealand; ^2^ Health New Zealand – Te Whatu Ora, Nelson Marlborough Nelson New Zealand; ^3^ Health New Zealand – Te Whatu Ora, Counties Manukau Auckland New Zealand; ^4^ Health New Zealand – Te Whatu Ora, Te Toka Tumai Auckland New Zealand; ^5^ Te Kupenga Hauora Māori, Faculty of Medical and Health Sciences The University of Auckland Auckland New Zealand

**Keywords:** beliefs, compassion, delivery of health care, empathy, motivation

## Abstract

**Aims:**

To develop and preliminarily validate a measure of beliefs about compassion in health care and assess whether and which beliefs may predict compassion.

**Design:**

Pre‐registered cross‐sectional online survey study with a repeated‐measures vignette component.

**Method:**

Exploratory and Confirmatory Factor analyses were performed on a split sample of 890 healthcare professionals in Aotearoa New Zealand (NZ). Links with fears of compassion for others, burnout, trait compassion, compassion competency and ability and self‐efficacy were used to assess convergent and divergent validity. Linear mixed model regression analyses were used to assess relationships between beliefs and compassion. In writing this report, we adhered to the Strengthening the Reporting of Observational Studies in Epidemiology (STROBE) guidelines.

**Results:**

Four‐factor structure featuring three negative (compassion as harmful, not useful, draining) and one positive (compassion is important) type of beliefs was established. Confirmatory factor analysis indicated a good fit and subscales indicated good measures of validity. Internal consistency was achieved for the subset of beliefs (harmful, not useful). Regression analyses indicated negative effects of the belief that compassion is draining on caring, motivation to help and compassion overall; negative effects of the belief that compassion is not useful on the motivation to help and a positive effect of the belief that compassion is important on caring and compassion overall. There was no effect of beliefs that compassion is harmful on compassion measures.

**Conclusion:**

This report extends prior qualitative studies of beliefs about compassion in a large healthcare sample, offering a way to measure these potentially malleable factors that might be targeted in education, interventions and future research.

**Patient or Public Contribution:**

The study was designed in consultation with healthcare and compassion research professionals, including substantial input from Indigenous Māori healthcare professionals.

**Summary**

- What does this paper contribute to the wider global clinical community?
  ○ Beliefs about compassion in health care have a complex structure but can be measured.
  ○ Beliefs about compassion in healthcare predict healthcare professionals' caring and motivation to help—the two core components of compassion.
  ○ The compassion beliefs scale presented in this report can be used to assess healthcare professional beliefs, identifying targets for intervention.

## 1 Introduction

Compassion is defined as a sensitivity to suffering together with a commitment to try to alleviate and prevent it (Gilbert et al. 2017). It plays a crucial role in health care and forms an integral part of medical ethics (Canadian Medical Association 2018; New Zealand Medical Association 2021). Compassion serves as a driving force in the pursuit of healthcare careers (Goel et al. 2018; Wu et al. 2015) and predicts better outcomes for patients, providers and organisations (Lains, Johnson, and Johnson 2024; Trzeciak, Roberts, and Mazzarelli 2017). Regrettably, compassion in health care can be inconsistent or even absent at times (Malenfant et al. 2022; Zandbelt et al. 2007). A lack of compassion has been linked to adverse medical events, more patient complaints and greater costs (Malenfant et al. 2022; Trzeciak, Roberts, and Mazzarelli 2017). Compassion is frequently treated as a moral value rather than systematically studied (Marshman et al. 2024; Pavlova and Consedine 2022), but it is increasingly clear that empirical investigation of the factors influencing compassion in health care is needed.

Both theoretical and empirical work indicate that there is likely no single reason why compassion might be challenged in healthcare (Fernando and Consedine 2014a; Pavlova et al. 2021; Wang et al. 2022). Although research has historically focused on static (and largely immutable) individual factors (e.g., occupation, gender, or professional experience) (Pavlova et al. 2021; Wang et al. 2022), more recent work have begun to consider the importance of organisational and cultural influences (Pavlova et al. 2023; Roskvist 2023). Indeed, reviews suggest that to be effective interventions must be considered within organisational contexts (Sinclair et al. 2020; Sinclair, Harris, et al. 2021). Likewise, theoretical perspectives suggest that insights into compassion are found at the *intersection* of various levels of influence—personal, relational and environmental (Fernando and Consedine 2014b; Pavlova and Consedine 2023).

Defined as a mental conviction about the truth or actuality of an idea (Schwitzgebel 2010), *beliefs* lie at the intersection of individual behaviour and workplace culture. Beliefs shape perceptions regarding the environment and represent a framework that guides action (Connors and Halligan 2015; Halligan 2006, 2007; Tullett et al. 2013). In other words, (organisational) culture and discourse that form one's beliefs matter (Lee et al. 2021; Nan, Wang, and Thier 2021). This is why inaccurate beliefs that may result in unhelpful behaviours are often the target of psychological interventions (Beck and Fleming 2021; Hofmann et al. 2012). As opposed to interventions solely targeting organisational cultures that might be difficult to change (Parmelli et al. 2011) or investing in individual‐focused knowledge and skills interventions that might fail to address underlying negative attitudes (Dempsey, McAlaney, and Bewick 2018; Sinclair et al. 2020; Sinclair, Harris, et al. 2021; Van Fleet and Griffin 2006), interventions that challenge unhelpful beliefs may be more effective (Hofmann et al. 2012).

The study of beliefs about compassion in health care is only just beginning. Work in community samples has linked *attitudes* towards compassion with the expression of compassion (Kirby et al. 2021). At the same time, although attitudes and beliefs are linked and may both influence behaviour (Fishbein 1966; Veilleux et al. 2021), they also differ in important ways. Specifically, attitudes represent the affective and the beliefs of the cognitive component (Millar and Tesser 1990), with the latter being more amenable to change (Hofmann et al. 2012) and also likely underpinning attitudes (Di Martino and Zan 2001). Systematic reviews (Pavlova et al. 2021; Wang et al. 2022) outline a growing body of qualitative evidence showing that healthcare‐specific beliefs about compassion may impact the expression of it. For example, believing that compassion can help achieve better treatment outcomes and make one's work more enjoyable predicts reports of greater compassion (Derksen et al. 2016; Swendiman et al. 2019; Uygur, Brown, and Herbert 2019). Conversely, believing that compassion negatively affects objectivity and professionalism, career progression or mental health predicts reports of lower compassion (Carmel and Glick 1996; Lyness 1993; Stratta, Riding, and Baker 2016). However, most studies regarding the possible importance of beliefs about compassion are qualitative, conducted in small, self‐selected samples and focus on specific beliefs. As such, quantitative work that increases content validity, capacitates robust tests of links to outcomes and permits commentary on the underlying structure of beliefs that may affect compassion in health care is clearly needed.

Given the importance of beliefs as potentially modifiable factors that predict compassion in health care, the current absence of measures, and good evidence that beliefs' are amenable to intervention, the primary and secondary aims of this report were (1) to develop and preliminarily validate a measure of compassion beliefs in healthcare and (2) to test whether beliefs predicted ratings of care and the desire to help (the key elements of compassion). Based on research on compassion attitudes in non‐healthcare samples (Kirby et al. 2021), our pre‐registration hypothesised a two‐factorial structure, with positive and negative beliefs predicting greater or lower compassion, respectively. Given the complex nature of compassion and the fact that patient care is a professional obligation, we also explore whether beliefs might be differentially associated with (a) *caring* as sensitivity to suffering and (b) the commitment or *motivation to help*. While studies often operationalise compassion globally, emerging evidence suggests that the “caring” and “helping” components of compassion might be impacted by different factors in healthcare (Fernando, Skinner, and Consedine 2017; Pavlova et al. 2024; Reynolds et al. 2019). We treat this as an exploratory question.

## 2 Materials and Methods

### 2.1 Design

This cross‐sectional report represents part of a broader study investigating the factors that influence compassion in healthcare. The study was designed in consultation with healthcare professionals, including substantial input from indigenous Māori healthcare professionals. Study procedures are reported elsewhere (Pavlova et al. 2023, 2024). The study was approved by the Auckland Health Research Ethics Committee (Approval Number AH23221) and independently by each of the 20 District Health Board localities in Aotearoa New Zealand. The study design, operationalisation and hypotheses were pre‐registered on AsPredicted (Registration number 75407: Hypothesis 2).

### 2.2 Participants and Setting

**FIGURE 1:**
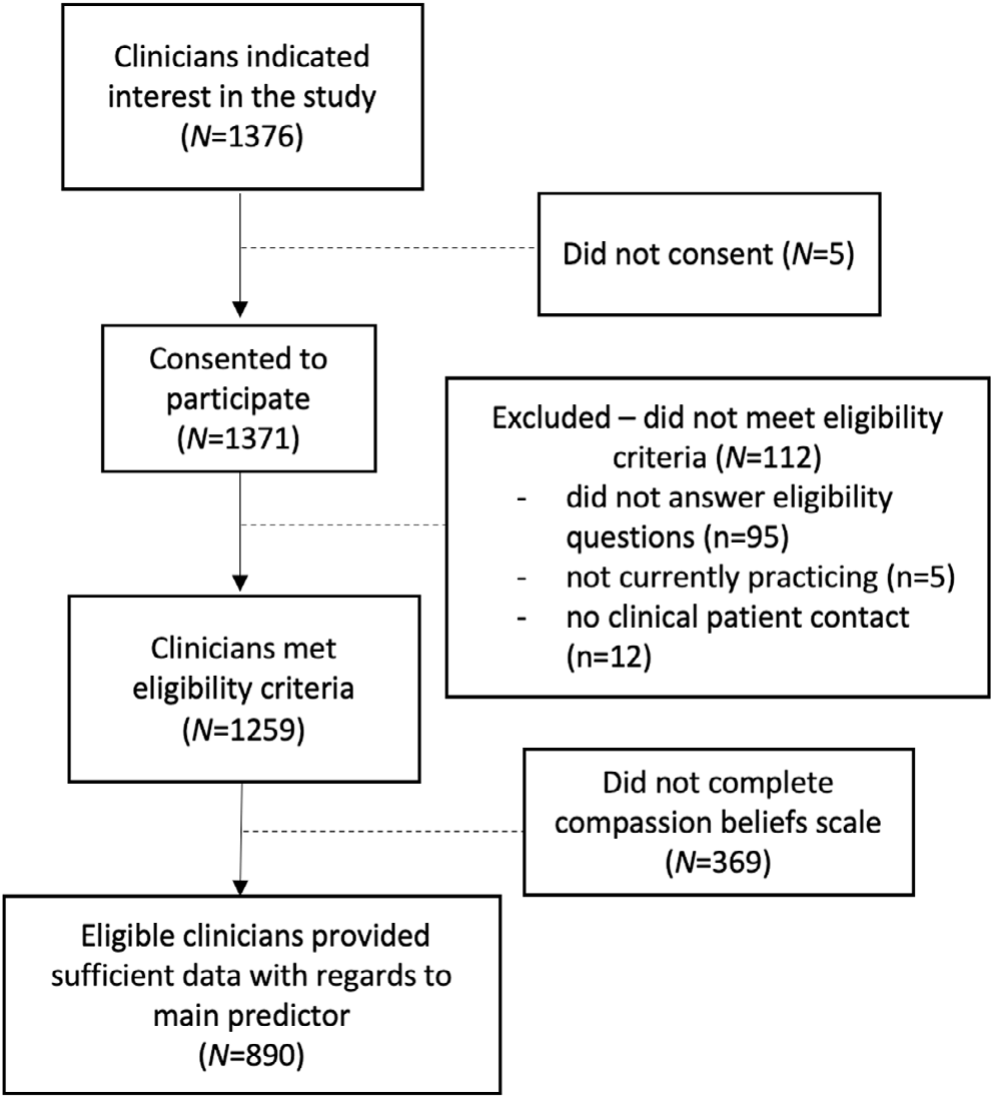
Recruitment flow diagram.

The study recruited part‐ or full‐time, English‐speaking healthcare professionals (doctors, nurses and allied health professionals such as social workers, psychologists, occupational therapists, sonographers, midwives, etc.) currently practicing in Aotearoa New Zealand in a patient‐facing role. Recruitment took place from February to May 2022 via organisational and professional unions' newsletters. The call to participate was also shared with health professional alumni from universities training health professionals and widely shared in professional networks. Participation was voluntary, informed and anonymous. The study was conducted via a 20‐min online survey. Of 1376 people initially responding, 1371 consented. In addition, 112 were excluded because they did not meet pre‐registration eligibility criteria (e.g., did not answer screening questions, were not currently practising, and/or reported no *clinical* patient contact). Of 1259 eligible participants, this report analysed data from the 890 participants completing the compassion beliefs scale (Figure 1). The sample size was sufficient to conduct Exploratory (EFA) and Confirmatory (CFA) Factor Analyses (Kyriazos 2018), reliability and validity measures (Frost et al. 2007) and to predict small effects in multi‐level regression analyses ([Cohen *f* ^2^ = 0.02 at 90% power for multiple regression with *N* = 890 conducted with 8 predictors at 0.05 significance level]) calculated via pwr package (Champely et al. 2018).

### 2.3 Materials and Measures

#### 2.3.1 Participants and Organisational Characteristics

Demographic and occupational data including gender, age and ethnicity, occupation and years of experience and organisational characteristics such as size (small/medium < 250 employees, large > 250 employees), funding source (private or public), operation within a cultural framework and setting (community/primary vs. secondary/tertiary; urban vs. rural) were collected.

#### 2.3.2 Primary Predictor—Compassion Beliefs in Healthcare Measure (CB‐H)

Broadly, the scale was developed in accordance with the stages of development outlined by Gehlbach and Brinkworth (2011). After determining the construct in question (i.e., beliefs) (1) and ascertaining that no prior scales existed to measure compassion beliefs in health care (2), the initial items developed for the CB‐H were created (3) based on studies captured within recent systematic reviews (Pavlova et al. 2021; Wang et al. 2022). These reviews captured 17 qualitative (*N* = 9–351), three mixed methods (*N* = 25–151) and two quantitative (*N* = 202–308) reports and included a range of disciplines and specialties conducted globally. In line with pre‐registration, items were designed to capture positive and negative compassion beliefs and featured beliefs about compassion in relation to oneself, patients, organisational contingencies, clinical outcomes and more general heuristics (e.g., “Being compassionate is hard”). Additional items and item wording were identified and adapted through conversation with healthcare professionals (doctors, nurses and allied health professionals) of Māori and New Zealand European ethnicity and diverse gender (4). Additional items regarding compassion potentially competing with excellence and safety and/or being time intensive were added after this process. The resultant 14 items were measured on a 5‐point Likert scale ranging from 1 = *Strongly disagree* to 5 = *Strongly agree* (Appendix S1.). The scale was then piloted in a group of 13 trainee health professionals, with the group indicating good comprehension of the scale and the item wording and the items showing a good level of variability (5).

### 2.4 Validation Measures

#### 2.4.1 Measures of Convergent Validity

The Fears of Compassion Scale—Fear of Compassion for Others and Burnout were used as related constructs to measure convergent validity. Theories of appraisal (Scherer and Johnstone 2001) suggest that emotions, attitudes, and beliefs are related, and beliefs are regarded as one of the key influences on emotions and attitudes (Frijda, Manstead, and Bem 2000). Thus, it was expected that Fears of Compassion for Others and Compassion Beliefs in Healthcare should be related. Regarding burnout, early evidence has shown that compassion and burnout (i.e., “a multifaceted condition of overwhelming exhaustion, interpersonal detachment or cynicism toward one's job, and a sense of reduced professional efficacy, driven by long‐term workplace stress” (Hewitt et al. 2020)) are inversely related (Pavlova et al. 2023). As such, Burnout was selected as a measure that could also likely be related to the Compassion Beliefs in Healthcare.

#### 2.4.2 Fears of Compassion Scale—Fear of Compassion for Others

Fears of Compassion for Others scale indices fears that compassion for others will result in negative consequences as well as the associated avoidance, indexing a generally negative compassion attitude (Gilbert et al. 2011; Kirby et al. 2021). Developed in general population samples, this 10‐item measure uses a 5‐point Likert scale (0 = Do not agree at all, 4 = Completely agree). The scale predicts lower trait compassion and greater anxiety (Gilbert et al. 2011). Internal reliability in the current and original studies were 0.89 and 0.84, respectively, similar to that in other healthcare samples (Matos et al. 2023).

#### 2.4.3 Burnout

Burnout was assessed using the 2‐Question Summative Score to minimise attrition (Li‐Sauerwine et al. 2020). Participants rated the following two items based on the Maslach Burnout Inventory—“I feel burned out from my work” and “I have become more callous toward people since I took this job”—using a 1 (*Never*) to 5 (*Always*) Likert scale. This two‐item scale has shown good convergent validity with Emotional Exhaustion (0.81) and Depersonalisation (0.73) in a sample of 1522 Emergency Medicine residents, with sensitivity and specificity of 93.6% and 73.0%, respectively. Summative scores have been widely used in healthcare samples. The correlation between the two items in the current sample was moderate (*r* = 0.47, *p* < 0.01).

#### 2.4.4 Measures of Divergent Validity

To index divergent validity, Compassion Ability, Compassion Competence, Trait Compassion and General Self‐Efficacy were selected as constructs that would likely be distinct from the Compassion Beliefs in Healthcare as we hypothesised that the beliefs would unlikely affect one's abilities, competencies, traits and/or self‐efficacy ratings.

#### 2.4.5 Compassion Ability (SCQ‐HCPASA)

Health professionals' self‐reported ability to express compassion was assessed using the 15‐item Sinclair Compassion Questionnaire—Healthcare Provider Ability Self‐Assessment adapted from the patient‐focused Sinclair Compassion Questionnaire (SCQ) (Sinclair, Hack, et al. 2021). The scale shows convergent validity with measures of trait compassion and self‐efficacy in New Zealand samples (Pavlova et al. 2023). This ability measure asks clinicians to rate how often they were able to show compassion (e.g., showing genuine concern, trying to understand patients' needs) on a 5‐point Likert scale ranging from 1 = *Never able* to 5 = *Always able*. Items were highly reliable (*α* = 0.95).

#### 2.4.6 Compassion Competence (SCQ‐HCPCSA)

Clinicians' self‐reported compassion competence was assessed by the Sinclair Compassion Questionnaire—Healthcare Provider Competence Self‐Assessment. In a manner similar to the SCQ Ability Scale, clinicians rate how competent they feel in their compassion skills on a 5‐point Likert scale ranging from 1 = *Not at all competent* to 5 = *Completely competent* and convergent validity data for New Zealand samples are available (Pavlova et al. 2023). The reliability of the scale was high (*α* = 0.94).

#### 2.4.7 Trait Compassion

Trait/dispositional compassion was measured by the Compassionate Love Scale‐Short Form (CLS‐H‐SF), stranger‐humanity version (Chiesi, Lau, and Saklofske 2020; Williams et al. 2022). The scale measures the tendency to experience compassion towards a stranger or greater humanity by assessing caring and motivation to help. The scale has shown convergence with empathy, helpfulness, volunteerism and spiritual experiences although there are some construct concerns (Strauss et al. 2016). Items are rated on a 7‐point Likert‐type scale (1 = not at all true of me, 7 = very true of me). Items are highly reliable in health care (Williams et al. 2022) and the current sample (*α* = 0.91).

#### 2.4.8 General Self‐Efficacy Scale (GSE‐6)

GSE‐6 asks participants to rate the extent to which each statement applies to them using a 4‐point Likert scale (1 = not true at all; 4 = exactly true) (Romppel et al. 2013). Because self‐efficacy might be expected to correlate with negative beliefs about compassion (Babenko and Oswald 2019) and relate to positive and negative affectivity (Luszczynska, Scholz, and Schwarzer 2005), the GSE‐6 was added as a measure of divergent validation. The short‐form scale internal consistency ranges from 0.79 and 0.87 (Romppel et al. 2013), including in healthcare samples (Sommaruga et al. 2017). Internal reliability in the current sample was 0.84.

#### 2.4.9 Primary Outcome

A face‐valid measure previously used in healthcare samples (Fernando, Skinner, and Consedine 2017; Reynolds et al. 2019) was used to capture the two core elements of compassion in health (i.e., the awareness of suffering and the motivation to help (Gilbert 2019; Jazaieri et al. 2013; Strauss et al. 2016)). Participants were asked how *caring* they felt towards patients depicted in two difficult patient vignettes (Pavlova et al. 2024) and the extent to which they would *want to help* using a 0–100 VAS scale (0 = *Not at all* and 100 = *Extremely*). Although caring and motivation to help were moderately to strongly correlated (*r* = 0.69–0.76, *p* < 0.05), we analysed caring and motivation to help components independently as well as the aggregate measure because these components have shown distinct links with other constructs in studies conducted in healthcare settings (Fernando, Skinner, and Consedine 2017; Pavlova et al. 2024; Reynolds et al. 2019).

#### 2.4.10 Social Desirability

Because ratings regarding compassion are prone to desirability bias in healthcare settings (Fernando, Arroll, and Consedine 2016), the Marlowe Crowne Social Desirability Scale short form version C (MC‐C) was administered (Ii and Sipps 1985). The MC‐C is a 13‐item, true/false measure where higher scores indicate a greater tendency towards providing socially desirable responses (Reynolds 1982). Correlated with the full measure (*r* = 0.91), the scale shows good test–retest reliability and adequate convergent validity in healthcare research. In the present sample, the scale reliability was moderate/low (*a* = 0.67), which is in line with what has been observed in prior healthcare samples (Baguley, Pavlova, and Consedine 2022; Fernando, Arroll, and Consedine 2016).

#### 2.4.11 Data Analysis

All analyses were performed in R. Data management decisions conducted prior to analyses were conducted in accordance with our pre‐registered analytic plan and are described in detail elsewhere (Pavlova et al. 2023, 2024).

Structural analyses of the Compassion Beliefs in Healthcare Scale were conducted via EFA and CFA on a split‐sample using the Fa method (Principal Access Factoring method, which is a data reduction technique to extract common factors or subscales, with an oblimin rotation, which allows for the factors to be correlated) for the EFA and lavaan package (Rosseel 2012) for the CFA. The sample was split according to a SOLOMON script specifically designed for factor analysis (Lorenzo‐Seva 2022) and the SemPaths package was used for model visualisation. Pearson's correlation analyses and comparison of correlations by confidence intervals (CI) (Zou 2007) were used for scale validation.

Because data were multilevel (Magezi 2015), the links between beliefs and indices of compassion were tested using stepped linear mixed model (LMM) analyses. LMM is increasingly used to analyse repeated‐measures data in experimental designs as it allows for the specification of random effects (i.e., clinicians) rather than bundling level‐associated variance into an error term (Kaburu et al. 2016). Analyses were run using lme4 package (Bates et al. 2009). Significance was calculated using the lmerTest package (Kuznetsova, Brockhoff, and Christensen 2017), which applies Satterthwaite's method to estimate degrees of freedom and generate p‐values for mixed models. Statistical significance was set at *p* < 0.05 for all tests, and 95% CI were reported. The effect sizes represent small (*f* ^2^ ≥ 0.02), medium (*f* ^2^ ≥ 0.15), and large effects (*f* ^2^ ≥ 0.35) (Selya et al. 2012).

The model specification was as follows:

$$\begin{array}{c} \text{Compassion}\sim {\text{Belief}}_{i}+\text{Gender}+\text{Occupation}+\text{Years of Experience}\\ \;+\text{Social Desirability}+\left(1|\text{Clinician}\right) \end{array}$$

A stepped regression approach was chosen to be able to separate out the effects of beliefs vs. confounders on compassion rather than merging the variation with other influences. In the first step, the compassion beliefs were entered. In the second step, the beliefs that showed significance were entered together with confounds. In line with the study pre‐registration, confounds were selected when (a) prior evidence suggested an association with the outcome (see Introduction) and (b) a univariate association with the predictor was in evidence (Appendix S2).

## 3 Results

### 3.1 Participants

Sample characteristics are presented in Table 1.

**TABLE 1:** Sample characteristics (*N* = 890).

	*N* (%)	*N* (%)
	M (SD)	Missing
Socio‐demographic characteristics		
Gender		—
Male	136 (15.5%)	
Female	748 (84.0%)	
Non‐binary	5 (0.5%)	
Ethnicity		—
New Zealand European	493 (55.4%)	
Māori	131 (14.7%)	
Asian	112 (12.6%)	
Pacific People	20 (2.2%)	
Middle Eastern, Latin American and African (MELAA)	16 (1.8%)	
Other ethnicity	117 (13.1%)	
Age	43.0 (13.0)	—
Dispositional measures		
Social desirability	21.17 (2.66)	16 (1.8)
Trait compassion	3.86 (0.68)	—
Self‐efficacy	4.16 (0.58)	12 (1.4)
Fear of Compassion from Others	25.74 (8.22)	7 (0.8)
Occupational characteristics		
Occupation		—
Doctors	184 (20.7%)	
Nurses	418 (47.0%)	
Allied and midwives	288 (32.3%)	
Years of experience	17.55 (12.81)	2 (0.2)
Brnout	5.58 (1.70)	—
Compassion competence	4.65 (0.37)	4 (0.5)
Ability to show compassion	4.08 (0.58)	16 (1.8)
Organisational characteristics		
Orgnaisational size (large > 250 vs. small)	675 (75.8%)	—
Funding type (public vs. private)	791 (88.9%)	—
Care type (primary vs. secondary)	299 (33.6%)	—
Urban (vs. rural)	788 (88.5%)	—
Belonging to cultural framework	482 (54.2%)	3 (0.3)
Beliefs about compassion subscales		
Harmful	3.12 (0.87)	—
Not useful	1.97 (0.81)	—
Draining	2.67 (1.02)	—
Important	4.11 (0.86)	—

### 3.2 The Structure of Compassion Beliefs in Healthcare

#### 3.2.1 Exploratory Factor Analyses

The results of this factor analysis are presented in Table 2. Four factors were extracted:

- Compassion is harmful to professionalism and mental health
- Compassion is not useful
- Compassion is draining
- Compassion is a professional responsibility and gives meaning

**TABLE 2:** Exploratory factor analysis (*N* = 890).

Items	M (SD)	Factor				Dimension	*α*
		1	2	3	4		
Too much compassion can negatively affect objectivity	3.37 (1.10)	**0.67**	−0.06	0.05	0.02	Harmful	0.71
Compassion can hinder professionalism	2.36 (1.11)	**0.67**	0.15	0.01	0.05		
Being too compassionate can negatively affect my own mental health	3.63 (1.10)	**0.47**	−0.11	0.40	−0.07		
Practicing with compassion will not help in professional career growth	1.89 (0.99)	−0.10	**0.67**	0.14	0.05	Not useful	0.7
Compassion competes with clinical excellence	1.95 (1.02)	0.02	**0.59**	0.06	0.02		
Being compassionate is unproductive towards patients who are emotive, biased or have unrealistic views	2.07 (1.04)	0.24	**0.58**	−0.01	0.02		
Compassion requires too much emotional work	2.49 (1.11)	0.04	0.14	**0.65**	0.10	Draining	0.63
Being compassionate is hard	2.85 (1.26)	0.00	0.07	**0.61**	−0.01		
Compassion is clinicians' professional responsibility	4.01 (0.93)	−0.03	−0.11	0.10	**0.65**	Important	0.51
Expressing compassion can give work meaning	4.13 (0.79)	0.03	0.15	−0.02	**0.49**		
Being compassionate will not solve medical problems	2.52 (1.10)	0.35	0.24	−0.08	0.15		
Safety should always come before compassion	3.55 (1.10)	0.28	0.28	0.04	−0.05		
There is often too little time for compassion	3.32 (1.26)	0.11	−0.01	0.35	0.01		
Compassion positively affects clinical outcomes	4.23 (0.87)	0.15	0.18	−0.18	0.39		
Eigenvalue		1.67	1.65	1.29	1.00		
Variance explained		0.12	0.12	0.09	0.07		

*Note:* Extraction method: PFA; Rotation method: Oblimin. Loadings larger than 0.40 are in bold.

The Kaiser‐Meyer‐Olkin measure showed sampling adequacy for the analysis (KMO = 0.84). Bartlett's test of sphericity showed significance (*χ* ^2^(91) = 1425.95, *p* < 0.001). The Principal Axis Factoring (pa) analysis with a cut‐off point of 0.40 and Kaiser's criterion of eigenvalues greater than 1 (see Field, Miles & Field 2012; Stevens 2002) yielded a four‐factor solution as the best fit for the data, accounting for 40% of the variance.

#### 3.2.2 Confirmatory Factor Analyses

**FIGURE 2:**
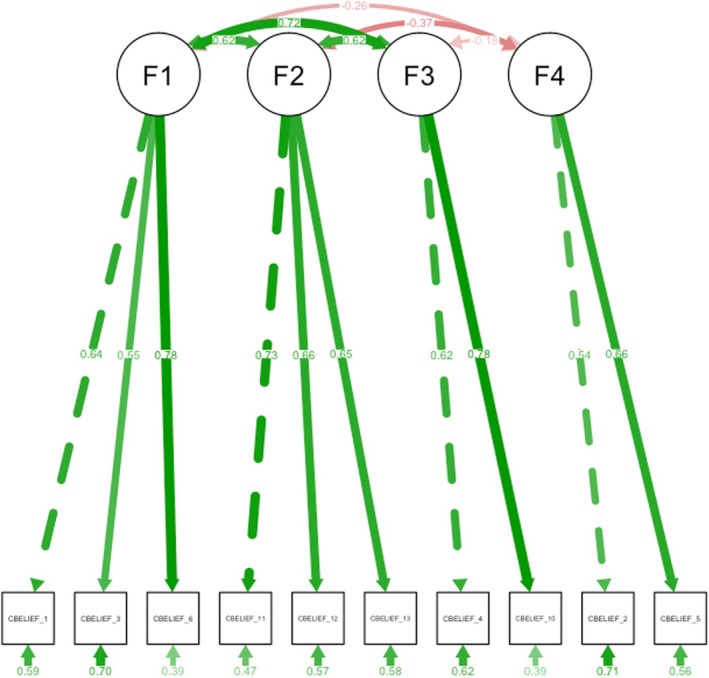
Confirmatory factor analysis model (*N* = 890). [Colour figure can be viewed at wileyonlinelibrary.com]

The four‐factor hierarchical model was replicated on the second part of the split sample, indicating good fit with the exemption of the Chi‐square statistic, likely due to the sensitivity of the Chi‐square statistic to large sample sizes (CFI = 0.92, RMSEA = 0.07, SRMR = 0.05, *χ* ^2^ = 112.23, df = 29, *p* < 0.001, AIC = 11985.25, BIC = 12091.74) (Hu and Bentler 1999; Kline 2023; Kyriazos 2018). The CFA model is visually presented in Figure 2.

#### 3.2.3 Tests of Convergent/Divergent Validation

Correlations with measures of compassion, dispositional social desirability, age and years of experience are presented in Table 3. Beliefs about compassion being harmful, not useful, and draining showed moderate positive associations with Fears of Compassion for Others and a weak positive association with Burnout. The beliefs that compassion is harmful *r*_Δ_(CI) = 0.04 (0.001–0.08) or not useful *r*_Δ_(CI) = 0.06 (0.02–0.10) were more strongly correlated with fears of compassion than was the belief that compassion is draining. Conversely, the belief that compassion is draining was more closely correlated with greater burnout than were the beliefs that compassion is harmful *r*_Δ_(CI) = 0.09 (0.05–0.14) or not useful *r*_Δ_(CI) = 0.14 (0.10–0.19); the belief that compassion is harmful was also more closely linked with greater burnout than the belief that compassion is not useful *r*_Δ_ (CI) = 0.05 (0.005–0.10). Thus, beliefs about compassion are (a) related to both fears of compassion and burnout as well as (b) distinct from one another.

**TABLE 3:** Correlation analyses (*N* = 890).

	Harmful	Not useful	Draining	Important
Convergent validation				
Fears of Compassion towards Others	0.521***	0.538***	0.481***	−0.145***
Burnout	0.252***	0.199***	0.344***	−0.007
Divergent validation				
Trait compassion	−0.225***	−0.199***	−0.319***	0.361***
Compassion competency	−0.128***	−0.175***	−0.291***	0.138***
Compassion ability	−0.140***	−0.154***	−0.291***	0.138***
Self‐efficacy	−0.121***	−0.086***	−0.215***	0.093***
Compassion measures				
Caring	−0.197***	−0.195***	−0.283***	0.173
Motivation to help	−0.132***	−0.150***	−0.231***	0.081***
Anticipated compassion	−0.177***	−0.185***	−0.276***	0.137***
Dispositional measures				
Social desirability	−0.159***	−0.026	−0.223***	−0.046
Other characteristics				
Years of experience	−0.189***	−0.051*	−0.093***	−0.093***
Age	−0.225***	−0.044	−0.165***	−0.109***

*p < 0.05, ***p < 0.001.

In terms of divergent validity, the belief scales showed negative, albeit weak, links with trait compassion, compassion ability and competence and general self‐efficacy (Table 3). The belief that compassion is draining was more strongly correlated with trait compassion, compassion competency, compassion ability and self‐efficacy than were the beliefs that compassion is harmful *r*_Δ_(CI) = 0.09 (0.05–0.14), *r*_Δ_(CI) = 0.14 (0.10–0.19), *r*_Δ_(CI) = 0.16 (0.12–0.21), *r*_Δ_(CI) = 0.09 (0.05–0.14) or not useful *r*_Δ_(CI) = 0.12 (0.07–0.17), *r*_Δ_(CI) = 0.13 (0.08–0.18), *r*_Δ_(CI) = 0.12 (0.07–0.17), *r*_Δ_(CI) = 0.13 (0.08–0.18). The belief that compassion is important showed weak positive correlations with trait compassion, compassion ability and competency, and general self‐efficacy and a weak negative correlation with Fear of Compassion for others, but not Burnout.

#### 3.2.4 Regression

The results of regression analyses are presented in Table 4. The overall model predicting *caring* explained 49.80% of the variance in caring, of which 14.2% of the variance was explained by the fixed effects. A stronger belief that compassion is draining (Cohen's *f* ^2^ = 0.05) predicted less caring towards a difficult patient after controlling for confounders while a greater belief in compassion's importance (Cohen's *f* ^2^ = 0.03) predicted greater compassion.

**TABLE 4:** Stepped linear mixed model (LMM) multivariate regression analyses (*N* = 890).

	Caring				Motivation to help				Compassion			
	Step 1		Step 2		Step 1		Step 2		Step 1		Step 2	
	*B*(CI)	*p*	*B*(CI)	*p*	*B*(CI)	*p*	*B*(CI)	*p*	*B*(CI)	*p*	*B*(CI)	*p*
(Intercept)	79.51	< 0.001	51.91	< 0.001	91.82	< 0.001	77.32	< 0.001	171.33	< 0.001	117.95	< 0.001
Fixed effects												
Negative compassion beliefs												
Harmful	−1.35 (−2.85 to 0.15)	0.08			−0.22 (−1.57 to 1.41)	0.76			−1.56 (−4.23 to 1.11)	0.25		
Not useful	−1.38 (−2.97 to 0.21)	0.09			−1.46 (−2.90 to −0.02)	0.04	−2.39 (−3.73 to −1.06)	< 0.001	−2.84 (−5.68 to 0.01)	0.05		
Draining	−4.57 (−5.86 to −3.30)	< 0.001	−4.73 (−4.82 to −2.24)	< 0.001	−3.91 (−5.07 to −2.76)	< 0.001	−2.99 (−4.09 to −1.90)	< 0.001	−8.49 (−10.78 to −6.21)	< 0.001	−8.29 (−10.30 to −6.28)	< 0.001
Important	3.69 (2.04 to 5.34)	< 0.001	4.66 (2.35 to 5.59)	< 0.001	1.05 (−0.44 to 2.54)	0.17			4.74 (1.80 to 7.68)	0.002	6.67 (2.87 to 9.48)	< 0.001
Covariates												
Gender (reference: female)			1.50 (−1.76 to 4.76)	0.37			1.50 (−1.44 to 4.43)	0.32			3.13 (−2.65 to 8.91)	0.29
Occupation: Nurse (reference: doctor)			9.95 (6.83 to 13.07)	< 0.001			8.84 (6.01 to 11.67)	< 0.001			18.37 (12.84 to 23.90)	< 0.001
Occupation: Allied (reference: doctor)			8.78 (5.49 to 12.06)	< 0.001			7.35 (4.37 to 10.32)	< 0.001			15.64 (9.81 to 21.47)	< 0.001
Years of experience			0.07 (−0.02 to 0.15)	0.35			0.08 (0.001 to 0.16)	0.04			0.16 (0.00 to 0.32)	0.05
Social desirability			0.32 (−0.09 to 0.77)	0.15			0.38 (0.001 to 0.78)	0.04			0.73 (−0.003 to 1.49)	0.06
Random effect												
Residual	174.9		160.1		94.09		80.53		481.1		426.9	
Variance (clinician level)	224.2	< 0.001	225.3	< 0.001	280.77	< 0.001	280.94	< 0.001	861.2	< 0.001	864.2	< 0.001
−2 Log‐likelihood	15,522.50		15,116.30		15,542.80		15,125.10		17,749.00		17,281.30	
Adjusted ICC	0.44		0.42		0.25		0.22		0.36		0.33	
R2 GLLM (c)	49.70%		49.80%		29.50%		29.70%		41.70%		41.80%	
R2 GLLM (m)	10.50%		14.20%		5.90%		7.30%		9.10%		13.10%	

The overall model for *motivation to help* as an outcome explained 29.7% of the variance, of which 7.3% of the variance was explained by the fixed effects. Greater beliefs that compassion is draining (Cohen's *f* ^2^ = 0.02) or not useful (Cohen's *f* ^2^ = 0.01) both predicted lower motivation to help after controlling for confounders, although effect sizes were modest.

The combined compassion model explained 41.7% of the variance, of which 13.1% was explained by the fixed effects. After controlling for confounders, a greater belief that compassion is too hard (Cohen's *f* ^2^ = 0.05) predicted lower compassion while a greater belief in compassion's importance (Cohen's *f* ^2^ = 0.03) predicted greater compassion.

## 4 Discussion

To our knowledge, this is the first study attempting to consolidate research and understand the structure of compassion beliefs in healthcare settings, as well as assess how such beliefs might predict compassion in healthcare. In contrast to related attitudinal research in general populations (and our pre‐registered hypothesis), both exploratory and confirmatory tests indicated that beliefs had a four‐factor rather than a two‐factor structure; initial validity analyses for the measure were promising. As importantly, most beliefs predicted ratings of compassion in response to difficult patient vignettes, although findings varied for ratings of care and motivation to help.

As such, given that beliefs are inherently malleable and may thus represent viable interventional targets, this report offers several implications. For example, given beliefs can be changed, evidence challenging unhelpful beliefs can be meaningfully integrated into healthcare education, both during initial training and during continuing professional development. Additionally, there might be a more subtle effect from health professionals familiarising themselves with the findings and implications of this report and becoming aware of their own compassion beliefs, reflecting on how these may facilitate or hinder the experience and expression of compassion.

### 4.1 The Structure of Beliefs About Compassion in Health care

As noted, beliefs about compassion represent a promising interventional target in healthcare settings. However, prior work examining beliefs has mostly relied on small‐scale qualitative studies and focused on idiosyncratically reported beliefs as potential barriers or facilitators of compassion (Pavlova et al. 2021; Wang et al. 2022), limiting insight into issues of belief's content and structure. Based on prior research and consultation with practising professionals, the scale tested here indexed a broad range of positive and negative beliefs, including beliefs about compassion concerning oneself and personal well‐being, the utility of compassion, its effects on patients and clinical outcomes and the role of organisational contingencies. Maximising content validity was a necessary first step to developing work and measurement in this field.

As discussed in the introduction, prior work in general population samples suggested that a two‐factor belief structure was likely. However, testament to the complexity of compassion in healthcare settings, our structural analyses indicated *four* different belief groupings. The fact that the structure of beliefs was more complex than expected may reflect the specific importance of compassion in healthcare settings and the range of influencing factors. Compassion in health care is professionally mandated (Paterson 2011), a core motivation for pursuing a caring profession (Goel et al. 2018; Wu et al. 2015), and is expected by professionals, patients and society at large (Malenfant et al. 2022; Trzeciak, Roberts, and Mazzarelli 2017). Compassion is thus highly salient in this setting; however, it may be challenged organisational demands that compete with compassion (Pavlova et al. 2023) or by implicit and explicit learning across the course of a professional career in which compassion is repeatedly deployed and challenged (Dev et al. 2019). By contrast, while compassion in the general population is morally desirable (Krettenauer et al. 2022), it mainly occurs towards the ingroup or kin or when a person directly encounters acute incidental suffering (Gilbert 2020; Kirby, Hoang, and Crimston 2024). Conversely, because compassion in health care is expected, remunerated, repeated, has a different carer‐to‐careé ratio and occurs in a clinical setting (Pavlova and Consedine 2022), it is inherently more complex, potentially contributing to the development of a more intricate set of related beliefs. Thus, while attitudes towards compassion might be almost definitionally positive or negative (Fishbein 1966), beliefs are more differentiated and amenable to intervention (Breckler and Wiggins 2014).

### 4.2 Evidence of Construct Validity

In addition to quantitatively assessing the structure of compassion beliefs, the current study was designed to offer preliminary evidence regarding the convergent and divergent validity of distinct compassion beliefs as well as assess their predictive validity; such questions are difficult to address using the qualitative methodologies that have predominated in prior research (Pavlova et al. 2021; Wang et al. 2022). Analyses presented here showed that “negative” beliefs about compassion tended to be associated with greater fears of compassion and greater burnout—both likely barriers to compassion in healthcare (Fernando and Consedine 2014a; Gilbert et al. 2011; Matos et al. 2023)—as well as less trait compassion, lower compassion ability and competency, and lower self‐efficacy—predictors of greater compassion (Babenko and Oswald 2019; Pavlova et al. 2023, 2021). Interestingly, a “positive” belief regarding the importance of compassion was not associated with burnout, perhaps indicating that positive compassion beliefs might not be protective or predictive of this occupational hazard.

Most directly, the results of this study showed that most beliefs about compassion do indeed predict compassion. To expand on this, first, likely due to the pervasiveness (Campbell 2020) and a potential priming effect (Roskvist 2023) of the “compassion fatigue” discourse (Fernando and Consedine 2014a; Ledoux 2015; Sinclair et al. 2017), the belief that compassion is draining showed the strongest effect, predicting lower overall compassion as indexed by ratings of both caring and motivation to help. Second, the belief that compassion is not useful, which we anticipate might stem from either (a) the lack of knowledge regarding positive compassion‐related outcomes (Trzeciak, Roberts, and Mazzarelli 2017) or (b) the fact that compassion is not a part of performance metrics (Pavlova et al. 2023), predicted lower motivation to help. Third, potentially grounded in health professionals' motivation for pursuing a career in health (Goel et al. 2018; Wu et al. 2015), the belief that compassion is important predicted greater caring and overall compassion.

Finally, and potentially related to the previous comment, there was a lack of an association between the belief that compassion is harmful and ratings of compassion. In other words, healthcare professionals might “feel the fear and do it anyway,” as it seems likely that a belief in compassion's importance may outweigh the healthcare push for objectivity or professionalism (Derksen et al. 2016). In other words, healthcare professionals are likely able to compromise some professional and personal outcomes in order to avoid distress from the inability to express compassion (Sinclair et al. 2017). However, they may also likely be fearful of being fatigued as a result of expressing compassion and seeing little use thereof.

This overall pattern and observations, together with the observation that the *magnitude* of the links between beliefs and convergent/divergent measures varied, offer greater confidence that the beliefs identified here are distinct from one another and may well have different origins. Perhaps more to the point, beliefs consistently predicted compassion ratings, implying that they may represent mutable targets for intervention in healthcare settings, especially considering that the intention to be compassionate is already present in healthcare samples (Ajzen 1991).

### 4.3 Distinct Links With Caring and Motivation to Help

Finally, in addition to understanding the structure of compassion beliefs and providing early evidence for the construct and predictive validity of a new measure, the final aim of this report was to assess whether caring and motivation to help might be differentially linked to beliefs. Prior studies show that caring and motivation to help respond differently to various external and internal stimuli. For example, a study assessing whether a brief mindfulness intervention facilitated compassion among medical students found that this intervention produced greater caring but did not impact motivation to help (Fernando, Skinner, and Consedine 2017). Similarly, another experimental study assessing the effects of clinicians' judgement of patients' responsibility for their suffering, showed greater effects on ratings of caring than motivation to help (Reynolds et al. 2019). Conversely, a recent study of clinical urgency suggested that urgency more strongly impacted the motivation to help rather than reports of caring (Pavlova et al. 2024). The current report contributes additional evidence to our understanding of compassion as a complex psychological phenomenon likely consisting of different, albeit not truly separable, psychological elements that respond differentially to different stimuli.

Specifically, except for the belief that compassion is draining (which was the strongest predictor for both components of compassion), the analyses presented here showed that not only did beliefs more robustly predict ratings of caring than they did motivation to help, but also that different beliefs differentially predicted these two components of compassion. Although we cannot be sure, our suspicion here is that the motivational processes underlying these two elements of compassion are distinct in healthcare settings, and thus, differentially linked to beliefs. In some views, for example, individuals strive to maximise the realisation of their goals (i.e., incentive) as a function of the probability of attainment of such goals (i.e., expectancy) (Beckmann and Heckhausen 2022). In compassion, it may be that caring is more driven by the “feeling” of compassion as an intrinsic motive, while “helping” is strongly influenced by professional obligation and external contingencies. The motivation to help is high in healthcare samples, partly because it is a professional obligation (Paterson 2011; Pavlova and Consedine 2022); this obligation and motivation remain even under challenge or where beliefs might not be supportive of feelings of care. Conversely, affective responses (such as feelings of care) are likely to be more strongly impacted by burnout, depersonalisation, or other related phenomena (Ferreira, Afonso, and Ramos 2020; Julia‐Sanchis et al. 2019; Lown, Shin, and Jones 2019; Zenasni et al. 2012), as well as by beliefs. To this extent, the decision to try to change particular beliefs about compassion in healthcare may depend on what outcomes (care vs. motivation to help) we want to prioritise.

### 4.4 Strengths, Limitations and Future Research

The development and validation of the beliefs about compassion in healthcare measures provide a useful initial contribution to the understanding of the nature of beliefs as well as to how and which of those beliefs might affect anticipated caring and motivation to help. Drawing from scattered qualitative studies, the study's strength lies within the careful scale development process and the quantitative testing in a large and diverse healthcare professional sample. Confidence in the results is increased by testing within a methodologically robust split‐sample process (which showed the stability of the structure of compassion beliefs in healthcare samples) as well as in using validated vignettes to gauge compassion (rather than “general” others) and the control over important confounds, including social desirability (Fernando, Arroll, and Consedine 2016).

These strengths noted that the study is not without its limitations. Most obviously, it should be recalled that the sample reflects a particular cultural and healthcare provision context and might not be generalisable. In other words, while struggling for compassion in healthcare seems widespread, it is possible that research in other settings might reveal slightly different findings. Second, our measures of care and the desire to help reflect responses to hypothetical vignettes rather than observation or drawing from patient ratings (Baguley, Pavlova, and Consedine 2022). While this should be borne in mind, it is also worth recalling that participants tend to respond similarly to hypothetical and real‐life scenarios similarly (Evans et al. 2015) and real‐life behavioural measures can be design‐expensive for the early stages of research. Third, considering the use of self‐report measures, the possibility of response bias could not be ruled out. Consistent with other work indicating that desirability bias is a pernicious problem in compassion in healthcare research (Fernando, Arroll, and Consedine 2016), we administered a social desirability measure; the correlation between social desirability and the beliefs' measure was low. Finally, although overall model performance indicated good measures of fit, the number of beliefs evidenced in structural analysis meant reliability was lower than would be desired for the “smaller” belief components. Low numbers of items tend to reduce scale reliability (Tavakol and Dennick 2011) and future studies would benefit from increasing the number of items for these smaller subscales. More broadly, it is possible that more than four types of beliefs about compassion exist. Indeed, four items related to clinical elements did not cleanly load and were thus removed. Again, informed by the structural insights offered here, future studies might consider adding these or other items into a revised version that is more comprehensive, even though the scale can be regarded as immediately usable.

## 5 Conclusion

Overall, this report presents early data from the development and analysis of a measure of compassion beliefs in healthcare settings. To our knowledge, this is the first study attempting to consolidate research, offering a way of measuring beliefs about compassion in health care, considering the underlying structure, and preliminarily assessing how such beliefs might predict compassion in a large sample of healthcare professionals. While compassion research has struggled to find ways to increase compassion in health care, beliefs are inherently malleable and may thus represent viable interventional targets in healthcare settings. Put simply, how we communicate about compassion in healthcare matters. Analyses revealed multiple types of beliefs about compassion, most of which predicted variation in caring and the desire to help (as well as compassion in general) in multivariate testing. Future research should attempt to validate the measure in other geographies, improve this initial measure by generating additional items associated with the types of beliefs, triangulate the results using behavioural observation or via patient ratings, and start considering how such beliefs might be challenged or changed. In addition, future studies may look to identify the origins of unhelpful beliefs about compassion and address such beliefs with educational interventions.

## Conflicts of Interest

The authors declare no conflicts of interest.

## Supporting information


Appendix S1.

Appendix S2.

## Data Availability

Due to the risk of the possibility of clinicians' reidentification, the data are not publicly available but can be made available upon request to the first author under certain conditions.
