# Supplementary material for: Feel the Fear and Do It Anyway—Beliefs About Compassion Predict Care and Motivation to Help Among Healthcare Professionals
Source: J Clin Nurs. 2024 Oct 24;34(7):2791–805. doi: 10.1111/jocn.17477 (PMC12181157; doi:10.1111/jocn.17477)
Supplement: Supplementary file 1 — Appendix S1. [file JOCN-34-2791-s002.pdf]

Appendix 1

The Compassion Beliefs in Healthcare scale (CB-H)

Instructions:
Please, indicate to what extent you agree with the following statements about compassion:

1. Too much compassion can negatively affect objectivity.

Strongly disagree	Somewhat disagree	Neither agree nor disagree	Somewhat agree	Strongly agree
○	○	○	○	○

2. Compassion is clinicians' professional responsibility.

Strongly disagree	Somewhat disagree	Neither agree nor disagree	Somewhat agree	Strongly agree
○	○	○	○	○

3. Being too compassionate can negatively affect my own mental health.

Strongly disagree	Somewhat disagree	Neither agree nor disagree	Somewhat agree	Strongly agree
○	○	○	○	○

4. Being compassionate is hard.

Strongly disagree	Somewhat disagree	Neither agree nor disagree	Somewhat agree	Strongly agree
○	○	○	○	○

5. Expressing compassion can give work meaning.

Strongly disagree	Somewhat disagree	Neither agree nor disagree	Somewhat agree	Strongly agree
○	○	○	○	○

6. Expressing compassion can give work meaning.

Strongly disagree	Somewhat disagree	Neither agree nor disagree	Somewhat agree	Strongly agree
○	○	○	○	○

7. Being compassionate will not solve medical problems.

Strongly disagree	Somewhat disagree	Neither agree nor disagree	Somewhat agree	Strongly agree
○	○	○	○	○

8. There is often too little time for compassion.

Strongly disagree	Somewhat disagree	Neither agree nor disagree	Somewhat agree	Strongly agree
○	○	○	○	○

9. Compassion positively affects clinical outcomes.

Strongly disagree	Somewhat disagree	Neither agree nor disagree	Somewhat agree	Strongly agree
○	○	○	○	○

10. Compassion requires too much emotional work.

Strongly disagree	Somewhat disagree	Neither agree nor disagree	Somewhat agree	Strongly agree
○	○	○	○	○

11. Being compassionate is unproductive towards patients who are emotive, biased, or have unrealistic views.

Strongly disagree	Somewhat disagree	Neither agree nor disagree	Somewhat agree	Strongly agree
○	○	○	○	○

12. Practicing with compassion will not help in professional career growth.

Strongly disagree	Somewhat disagree	Neither agree nor disagree	Somewhat agree	Strongly agree
○	○	○	○	○

13. Compassion competes with clinical excellence.

Strongly disagree	Somewhat disagree	Neither agree nor disagree	Somewhat agree	Strongly agree
○	○	○	○	○

14. Safety should always come before compassion.

Strongly disagree	Somewhat disagree	Neither agree nor disagree	Somewhat agree	Strongly agree
○	○	○	○	○

Appendix 2

Univariate analyses for the selection of confounding variables

Correlations with measures of compassion, dispositional social desirability, age, and years of experience are presented in Table 3 of the manuscript (see the table copied below). For categorical variables, analyses of variance showed that a belief that compassion is draining was lower among women relative to men ($F(1,883)=6.50$, $p=0.01$). No other beliefs showed differences by gender ($p>0.05$). Nurses ($p=0.02$) and allied health ($p<0.001$) also reported a lower belief that compassion might be draining than doctors ($F(1,883)=7.59$, $p<0.001$), although this pattern may reflect the fact that nurses ($p<0.001$) and allied healthcare professionals ($p<0.001$) were also more likely to be female (χ^2 (2)=250.57, $p<0.001$). With regards to ethnicity, Asian healthcare professionals reported a greater belief that compassion might not be useful ($F(5,884)=4.42$, $p<0.001$) and that it was draining ($F(5,884)=5.67$, $p<0.001$) than professionals who identified as Māori ($p<0.001$), New Zealand Europeans ($p<0.01$), or another ethnicity ($p<0.01$). No other differences in beliefs related to ethnicity were identified ($p>0.05$).

With regards to the organisational characteristics, healthcare professionals working privately reported a greater belief that compassion is harmful ($F(1,888)=11.81$, $p<0.001$) or was not useful ($F(1,888)=7.51$, $p<0.01$). Rural healthcare professionals reported a greater belief that compassion was not useful ($F(1,888)=10.52$, $p<0.001$) while urban professionals reported a greater belief in the importance of compassion ($F(1,888)=12.72$, $p<0.001$). Interestingly, healthcare professionals who worked for organisations affiliated with a cultural framework reported stronger beliefs that compassion is harmful ($F(1,885)=5.76$, $p=0.02$) and not useful ($F(1,885)=18.26$, $p<0.01$). There were no differences in beliefs associated with care setting (e.g. primary or secondary) ($p>0.05$) or organisational size ($p>0.05$).

	Beliefs			
	Harmful	Not useful	Draining	Important
Convergent validation				
Fears of Compassion towards Others	0.521***	0.538***	0.481***	-0.145***
Burnout	0.252***	0.199***	0.344***	-0.007
Divergent validation				
Trait compassion	-0.225***	-0.199***	-0.319***	0.361***
Compassion competency	-0.128***	-0.175***	-0.291***	0.138***
Compassion ability	-0.140***	-0.154***	-0.291***	0.138***
Self-efficacy	-0.121***	-0.086***	-0.215***	0.093***
Compassion measures				
Caring	-0.197***	-0.195***	-0.283***	0.173
Motivation to help	-0.132***	-0.150***	-0.231***	0.081***
Anticipated compassion	-0.177***	-0.185***	-0.276***	0.137***
Dispositional measures				
Social desirability	-0.159***	-0.026	-0.223***	-0.046
Other characteristics				
Years of experience	-0.189***	-0.051*	-0.093***	-0.093***
Age	-0.225***	-0.044	-0.165***	-0.109***

* <0.05; ** < 0.01; *** < 0.001
